# The regulation of emotions in adolescents: Age differences and emotion-specific patterns

**DOI:** 10.1371/journal.pone.0195501

**Published:** 2018-06-07

**Authors:** Anne Theurel, Edouard Gentaz

**Affiliations:** 1 SensoriMotor, Affective and Social Development Lab, Geneva, Switzerland; 2 University Grenoble Alpes, LPNC-CNRS, Grenoble, France; Southwest University, CHINA

## Abstract

Two experiments addressed the issue of age-related differences and emotion-specific patterns in emotion regulation during adolescence. Experiment 1 examined emotion-specific patterns in the effectiveness of reappraisal and distraction strategies in 14-year-old adolescents (*N* = 50). Adolescents were instructed to answer spontaneously or to downregulate their responses by using either distraction or cognitive reappraisal strategies before viewing negative pictures and were asked to rate their emotional state after picture presentation. Results showed that reappraisal effectiveness was modulated by emotional content but distraction was not. Reappraisal was more effective than distraction at regulating fear or anxiety (threat-related pictures) but was similar to distraction regarding other emotions. Using the same paradigm, Experiment 2 examined in 12-year-old (*N* = 56), 13-year-old (*N* = 49) and 15-year-old adolescents (*N* = 54) the age-related differences a) in the effectiveness of reappraisal and distraction when implemented and b) in the everyday use of regulation strategies using the Cognitive Emotion Regulation Questionnaire. Results revealed that regulation effectiveness was equivalent for both strategies in 12-year-olds, whereas a large improvement in reappraisal effectiveness was observed in 13- and 15-year-olds. No age differences were observed in the reported use of reappraisal, but older adolescents less frequently reported using distraction and more frequently reported using the rumination strategy. Taken together, these experiments provide new findings regarding the use and the effectiveness of cognitive regulation strategies during adolescence in terms of age differences and emotion specificity.

## Introduction

Emotion regulation can be defined as goal-directed processes functioning to influence the intensity, duration, and type of emotion experienced [1]. Emotion regulation has been related to a wide variety of domains of functioning, including social functioning, psychological and physical well-being, and academic performance (for review, see [2]). Despite the importance of emotion regulation in daily life situations, little is known about its development. Much of the research on emotion regulation focuses either on infancy and childhood or on adulthood. However, adolescence, a phase of gradual transition between childhood and adulthood (commonly considered to be the age range from roughly 12 to 18 years), is also relevant for emotion regulation because it is a period with fast and fundamental alterations in biological, cognitive, social, and emotional domains (e.g., [3–5]). Furthermore, adolescence has been associated with an increasing incidence of internalizing symptoms [6]. Studies suggest that the peak age of onset for having internalized symptoms is 14–15 years ([7–9]) and the majority of individuals who develop depression experience their first clinically significant episode during the transition from middle to late adolescence (i.e., ages 15–18 years). Such evidence argues for the need to identify vulnerability factors to depression in adolescence so that intervention efforts can be initiated prior to the surge in depression rates. Furthermore, according to Skinner and Zimmer-Gembeck [10], there is a significant shift in the nature and frequency of use of different emotion regulation strategies during the age period of early to middle adolescence (about ages 14–16). Given that emotion regulation is related to the development and maintenance of youth psychopathology (e.g., [11]), the transition period of early to middle adolescence seems to be a particularly informative period for studying the development of emotion regulation.

Many strategies have been identified for regulating emotional responses. The most prominent approach to organizing these strategies has been to focus on the time point at which regulatory processes are brought to bear on emotion-evoking situations. The modal model of emotion [1] specifies a sequence of processes involved in emotion generation: situation-attention-appraisal-response. Each process is a potential target for regulation strategies. This comprehensive and detailed model has received much empirical attention in the adult years.

Distraction and reappraisal are among the most powerful and the most studied forms of emotional regulation [12]. Distraction operates primarily through the use of attentional deployment and involves a shift in attention either away from emotional aspects of the situation or away from the situation altogether. Emotion-related information is regulated at an early processing stage via a filtering mechanism that prevents it from capturing selective attention. By contrast, reappraisal operates primarily through meaning-evaluation mechanisms that serve to compute and alter the affective significance of an emotional stimulus. Incoming emotional information that passes the early filter is regulated at a later stage via a second filtering mechanism that operates at the level of semantic meaning and determines the final output of the system. Although studies provide dissociations between reappraisal and distraction (for review, [13]), both have been shown to be effective ways to manage emotions (e.g., [12]) and to have a beneficial role with regard to internalized symptoms such as depression or anxiety (e.g., [14]). These two strategies appear to be particularly protective against the psychological symptoms that increase during the adolescent years.

However, the overview of literature gives rise to the debate about which specific strategy is most effective and whether these emotion regulation strategies have specific effects for different types of psychological symptoms and/or emotions. Indeed, while studies demonstrated negative relationship between use of reappraisal and anxiety and depression symptoms (e.g.,[15, 16]), distraction has been shown to reduce depressed affect [17, 18] but not anxiety (e.g., [19]). Furthermore, studies that compare the effectiveness of these two regulation strategies reveal a mixed picture according to the specific emotion being down-regulated (e.g., [20, 21]). To our knowledge, no study has tested the difference of effectiveness of these two strategies in adolescents. The first objective (Experiment 1) was to determine which specific strategy is most effective to downregulate negative affect in adolescents and whether these emotion regulation strategies have specific effects for different types of emotions. Given the link between emotions and emotional disorders (e.g., [22]) such study can provide evidence regarding which regulation strategy that should be targeted for specific psychological symptoms in mental health prevention and intervention programs for adolescents populations.

Furthermore, developmental differences in the effectiveness of regulation strategies are understudied, leaving questions regarding the importance and feasibility of coaching adolescents to use regulation strategies unanswered. Cognitively, high-level executive and social processes needed for emotion regulation, including working memory, inhibitory control, abstract thought, decision making, and perspective taking, all undergo development during adolescence (e.g.,[23–26]). Several studies have suggested that a higher level of cognitive resources might confer greater success at emotion regulation (e.g., [27]). During adolescence, there is increased cortical cognitive control over automatic sub-cortical emotional reactivity, possibly resulting in better emotional regulation [28]. Moreover, structural brain development in regions subserving emotion regulation continues over the course of adolescence (e.g.,[29]). Given this evidence, one could suppose that as adolescents mature, they have more efficient cortical functioning that result in more effective regulation of emotions. However, studies on the development of emotional regulation strategies in adolescence used different measurement approaches (e.g., observation, self-report, interviews, experience sampling or lab-based paradigm) and the empirical evidence regarding age differences reveals a mixed picture (e.g., [6]). Results in which a lab-based paradigm was used suggest development in reappraisal and distraction effectiveness [28, 30, 31]. However, studies on the self-reported use of regulation strategies reveal mixed results [27, 32, 33]. These diverging results suggest that development in emotion regulation effectiveness may not be paralleled by increasing spontaneous use with age in everyday life. There is a need to assess the effectiveness of implemented regulation strategies and their everyday use together in order to develop our understanding of the development of emotion regulation abilities in adolescence. The second objective (Experiment 2) was to determine if the distraction and reappraisal effectiveness and the daily use these strategies increases during adolescence. Such study can provide evidence on the effectiveness of regulation strategies at different ages in order to coach adolescents to use these strategies in their everyday life if it is not the case.

## Experiment 1: Does regulation effectiveness in adolescents depend on specific emotions?

Studies suggest that distraction and reappraisal may differ in their effectiveness to reduce internalized symptoms and to regulate specific emotions. Indeed, reappraisal has demonstrated clear efficacy for the anxiety symptoms reduction (e.g., [15]) and authors have found a robust negative relationship between self-reported use of reappraisal and depressive symptoms (e.g., [16]). In the same vein, distraction is an effective strategy to reduce depressed affect [17, 34] but evidence lacks regarding its effect on anxiety [e.g., 19]. In adolescents, reappraisal predicted lower levels of anxiety but showed no relations with sadness and the use of distraction predicted lower levels of sadness and is associated with the down-regulation of anxiety affect only among anxious youth [18]. Furthermore, studies that compare the effectiveness of these two regulation strategies reveal a mixed picture. Indeed, reappraisal seems to be more effective than distraction on anxiety [20, 35] but no difference were observed between these two strategies on sadness and depression [21, 36]. In adolescence, studies also suggest that regulation strategies effectiveness depends on specific emotions [37, 38]. But to our knowledge, no study has tested the difference of effectiveness of these two strategies in adolescents. The distinct mechanisms and dissociations between reappraisal and distraction (e.g., [13]) suggest that the effectiveness of these two strategies may differ in an emotion-specific manner. Given that, unlike distraction, reappraisal allows elaborated cognitive processing of emotional information at a semantic level, the effectiveness of the reappraisal strategy should be modulated by emotional picture content, whereas the effectiveness of the distraction strategy should remain stable across conditions. Although major studies on emotion regulation have used the International Affective Picture System (IAPS; [39]), this set of pictures elicits specific emotions, and fear has been the most elicited negative emotion [40]. It would be interesting to take account of other emotions to better understand emotion regulation strategies. Situations related to moral violations (e.g., human rights’ violation, animal mistreatment) are often emotionally charged. Emotions that are associated with moral violation may require cognitive regulation to diminish the unwanted emotional response. Whether regulation of emotions related to moral violations is similar to emotion regulation involving other emotional experiences, such as fear is yet unclear. One study in adults suggests different brain activation during regulatory processes depending on the specific emotional content involved (moral vs. non-moral situations): medial prefrontal cortex activity was augmented and amygdala activity was attenuated in the moral condition only [41]. Given these results, in Experiment 1 we select pictures related to moral violations (focusing on human rights’ violation and animal mistreatment) from the Geneva Affective Picture Database (GAPED; [42]) together with threat-related pictures from the IAPS. We aimed to compare the effectiveness of distraction and reappraisal strategies in 14-year-old adolescents and to examine how specific emotions affect both strategies effectiveness.

## Method

The present experiment was conducted in accordance with the Declaration of Helsinki and the educational organization of France. Informed consent was obtained from all participants included in the study. The ethics committee of the Laboratory of Psychology and Neurocognition from Grenoble-Alpes University and of the Faculty of Psychology and Education Sciences from the University of Geneva approved this study.

### Participants

Fifty-five 14-year-olds enrolled in the eighth grade in a French middle school were recruited. Written consent of the parents was obtained for each participant. Five participants (9% of the sample) were excluded from analyses because of technical problems (N = 2), refusal to terminate the experiment (N = 1) and after statistical analysis of outliers (N = 2). The final sample included 50 adolescents (20 girls, 30 boys) aged 13 years and 8 months on average (13 years and 2 months to 14 years and 6 months).

#### Stimuli

A total of 120 pictures (600 × 400 pixels) were taken from the IAPS, the GAPED, and a set of similar pictures that had been previously used in research with children [43]. One-half of this set of pictures (*N* = 60) was called “moral violation” and depicted human rights violations and animal mistreatment situations corresponding to low compatibility with moral norms. The other half (*N* = 60) depicted threat-related content (e.g., frightening animals, weapons, attacks). Moreover, regarding the effect of social content on regulation strategies [31], one-half (*N* = 60) of the set of pictures was composed of social pictures (depicting humans) and the other half (*N* = 60) of nonsocial pictures (depicting animals only). In sum, the set was composed of 30 social and 30 non-social moral-violation pictures and 30 social and 30 non-social threat-related pictures. To ensure the valence and arousal of pictures were equal to each other across the three instruction conditions (look, reappraisal, distraction), we created 3 sub-sets of pictures that were counterbalanced across regulation condition and participants. Furthermore, we check if the valence ratings in passive watching condition were equivalent across the three subsets of pictures. Results revealed no significant effect of pictures subsets, *F*(2, 27) = 1.74; *p* = .20.

Moral violation and threat-related pictures were controlled to prevent them from eliciting the same emotion. The set of pictures was pretested in 33 adults (mean age 32 years, 6 months, 22 women and 11 men). Participants watched each picture and were asked to determine which emotions that the picture made them feel by using the following responses: anxiety, anger, guilt, disgust, shame, contempt, fear, pity, sadness, or “any emotion.” We conducted multivariate analyses of variance (MANOVAS) on the percentage of responses per emotion, considering emotional picture content (moral violation, threat-related) as a between-subjects factor. The percentage of responses per emotion significantly differed between moral violation and threat-related pictures, *F*(10, 49) = 25.92, *p* < .001, η^2^ = 0.84. Tukey’s HSD test revealed significant differences for sadness (*p* < .01), pity (*p* < .01), guilt (*p* < .01), shame (*p* < .01), fear (*p* < .01) and anxiety (*p* < .01), but no differences between moral violation and threat-related pictures for anger (14% and 18%, respectively, *p* = .24), disgust (15% and 11%, *p* = .35), contempt (3% and 5%, *p* = .12), or any emotion (15% and 16%, *p* = .74). Moral violation pictures were more frequently rated as making a participant feel sadness (35%), pity (28%), guilt (14%), and shame (9%) than were threat-related pictures (1%, 1%, 4%, and 5%, respectively). By contrast, threat-related pictures were more frequently rated as making a participant feel fear (37%) and anxiety (23%) than were moral violation pictures (3% and 1%, respectively). Results also revealed that moral violation pictures were more frequently rated as making a participant feel sadness and pity than other emotions (all *p*’s< .01). Threat-related pictures were more frequently rated as making a participant feel fear and anxiety than other emotions (all *p*’s< .01).

### Procedure

Adolescents were tested individually in a quiet room within their school. First, participants were trained on task procedures. During the training phase, participants were told (1) to react naturally to the negative pictures presented after they read the cue “look”; (2) to tell themselves a story about the negative pictures that made them feel better (e.g., imagining that the picture belongs to a movie or imagining themselves standing farther away from the scene) when they read the cue “distance” (i.e., reappraisal); and (3) to search for geometrical shapes (e.g., square, cross) of different colors hidden in aversive pictures at a location judged to be neutral (non-emotionally arousing) after they read the cue “search for” (i.e., distraction). To ensure participants understood the regulation instructions and complied with the instructions during the whole experiment, we proposed several practice trials before participants take part in the experimental task. For instance, in reappraisal practice trials, participants were given examples of how to reappraise (e.g., imagining themselves standing farther away from the scene) and were asked to report their reappraisal strategy aloud.

**Fig 1:**
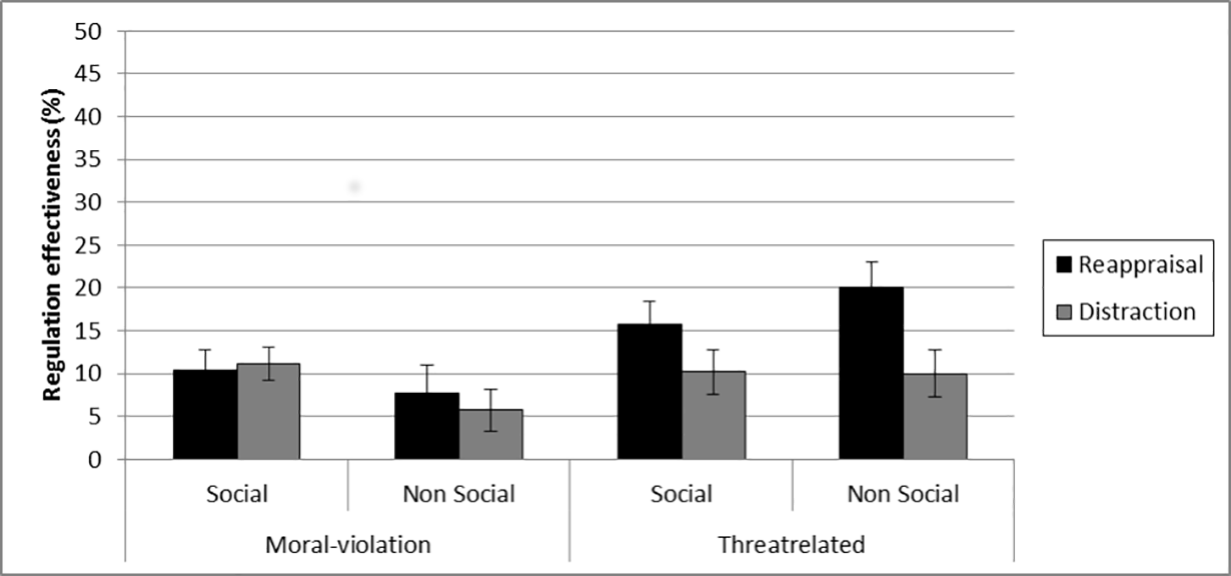
Regulation effectiveness score (%) as a function of regulation strategy, social content, and emotional content in 14-year-old adolescents. The error bars correspond to one standard error.

In the experimental session, participants watched 120 negative pictures (shown for 3 s) with three preceding 2-s displays: the item “distance” (the reappraisal condition; *N* = 40), the item “search for” (the distraction condition; *N* = 40), and the item “look” (the look or the non-regulation condition; *N* = 40). In the distraction condition, following the 3-s presentation of the picture, participants saw four geometrical shapes of different colors and were asked to respond by pressing a key to indicate which shapes were hidden in the picture just seen (Fig 1). In the look and reappraisal conditions, following the 3-s presentation of the picture, participants saw a black screen for 1 s. The pictures and the instructional cue were presented randomly. Three sets of 40 negative pictures were counterbalanced across participants with the look, distraction, and reappraisal conditions. After watching each picture and using the strategy indicated by the cue word, participants then rated their current strength of negative affect on a 5-point scale from 1 (*not feeling badly*) to 5 (*extremely badly*) by pressing a key. Stimuli were presented and button responses collected by using E-Prime 2.0 software.

## Results

### Affect ratings

Mean negative affect ratings were calculated for the look, reappraisal, and distraction conditions. Table 1 presents the mean affect rating according to the instruction condition (distraction, reappraisal, look), social content (social, nonsocial), and emotional content (moral violation, threat-related).

**Table 1:** Mean affect rating (and SD) according to instruction condition (distraction, reappraisal, look), social content (social, nonsocial), and emotional content (moral violation, threat-related).

		Instruction condition		
Emotion content		Distraction	Reappraisal	Look
Moral violation	Social	2.59 (0.81)	2.60 (0.83)	2.89 (0.71)
	Nonsocial	2.55 (0.89)	2.47 (0.89)	2.77 (1.00)
Threat-related	Social	2.78 (0.91)	2.61 (0.89)	3.10 (0.85)
	Nonsocial	2.60 (1.00)	2.32 (1.05)	2.93 (1.04)
Total		2.63 (0.82)	2.50 (0.83)	2.92 (0.79)

In order to determine whether the mean affect rating was equivalent for emotional and social content, we performed an ANOVA on the mean affect rating in the look condition (i.e., non-regulation), considering emotional content (moral violation, threat-related) and social content (social, nonsocial) as the within-subject factors. Results show that social pictures were rated as negatively higher (*M* = 3, *SD* = 0.72) than were nonsocial pictures (*M* = 2.95, *SD* = 0.90), *F*(1, 49) = 5.50; *p* < .05, η^2^ = 0.10, and that threat-related pictures were rated as negatively higher (*M* = 3.02, *SD* = 0.88) than were moral violation pictures (*M* = 2.83, *SD* = 0.78), *F*(1, 49) = 5.74; *p* < .05, η^2^ = 0.11. Interaction between emotional content and social content was not significant, *F*(1, 49) <1, *p* = .72.

### Regulation effectiveness

To check for a main effect of regulation (look > reappraisal and look > distraction), we conducted a preliminary analysis of variance (ANOVA) on these mean negative affect ratings, considering the instruction condition (reappraisal, distraction, look) as the within-subject factor. Results confirmed a main effect of the instruction condition, *F*(2, 98) = 33.33, *p* < .001, η^2^ = 0.53, showing that the mean negative affect rating was significantly higher in the look condition (*M* = 2.92, *SD* = 0.79) than it was in the reappraisal condition (*M* = 2.50, *SD* = 0.83, *p* < .001) or in the distraction condition (*M* = 2.63, *SD* = 0.82, *p* < .001), the difference between the latter being significantly different, *p* < .05.

As proposed by Silvers et al. [31], the regulation effectiveness score was then calculated as the percentage of negative affect that was decreased by reappraisal on distance trials in comparison to look trials ([look–distance]/look × 100) or by distraction on search for trials in comparison to look trials ([look–search for]/look × 100).

We conducted an ANOVA on regulation effectiveness, considering regulation strategy (reappraisal, distraction), emotional content (moral violation, threat-related), and social content (social, nonsocial) as the within-subject factors. Results showed a significant effect of regulation strategy, *F*(1, 49) = 5.80; *p* < .05, η^2^ = 0.11, revealing that regulation effectiveness was higher when participants implemented the reappraisal strategy (*M* = 13.50%, *SD* = 14.40%) than when they implemented the distraction strategy (*M* = 9.28%, *SD* = 11.02%). This regulation strategy effect significantly interacted with emotional content, *F*(1, 49) = 13.13; *p* < .01, η^2^ = 0.21. Tukey’s HSD test revealed that regulation effectiveness was higher with reappraisal (*M* = 17.94%, *SD* = 17.46%) than with distraction (*M* = 10.12%, *SD* = 14.97%) on threat-related pictures (*p* < .01), but that this difference was not significant on moral violation pictures (*M*^*R*^ = 9.05%, *SD*^*R*^ = 15.25%; *M*^*D*^ = 8.45%, *SD*^*D*^ = 11.26%; *p* = .97). Reappraisal was less effective with moral violation pictures than with threat-related pictures, *p* < .01, but no significant difference between emotional pictures was observed for distraction, *p* = .64.

The regulation strategy effect did not interact with social content, *F*(1, 49) = 1.79; *p* = .19. Other interactions were not significant (*F* < 1). Fig 1 presents the regulation effectiveness score as a function of regulation strategy, social content, and emotional content.

Finally to check if distraction was successfully used, we analyzed performance at the distraction task. With a mean percentage of correct response of 77.7%, results confirmed that participants successfully used distraction.

## Discussion

The main result revealed that reappraisal strategy is more effective than distraction in diminishing negative affect in adolescents, in line with the results observed in adults [12]. Such evidence emphasizes the need to target this strategy in mental health prevention and intervention programs for adolescents populations (as it is the case in cognitive behavioral therapy for instance, e.g., [44]).

Results also showed that the effect of the regulation strategy depends on specific emotional content, as reappraisal effectiveness is modulated by emotional content but distraction is not. This result corroborates previous findings suggesting that the effectiveness of specific regulation strategies varies depending on the targeted emotion in children [45], adults [46], and adolescents [37, 38]. This interaction can be explained by the distinct mechanisms underlying distraction and reappraisal. Indeed, distraction operates at an early processing stage via a filtering mechanism that does not allow elaborated cognitive processing of the emotional information [13]. Consequently, unlike reappraisal, distraction was not affected by the emotional content of the stimulus. By contrast, reappraisal regulates emotions via a filtering mechanism that operates at the level of semantic meaning and is consequently affected by the emotional content of the stimulus. Additionally, a study has shown that when processing emotions conveyed by moral situations, adults recruit brain regions associated to semantic knowledge while adolescents recruit brain regions associated to mentalizing [47]. The fact that adolescents, compared to adults, don’t rely on semantic knowledge when processing emotions conveyed by moral situations is a factor that can reduce reappraisal effectiveness in the case of moral violation pictures. Such findings can explain why reappraisal is less effective for moral violation than for threat related pictures.

However, of note is that threat-related pictures were rated as negatively higher than moral violation pictures in our experiment. The difference of emotion regulation effect could then result from the difference of valence strength between these two kinds of emotional stimuli. But according to [13] findings, reappraisal should have been more effective than distraction with emotional pictures of low intensity than with pictures of high intensity. The fact that reappraisal effectiveness was higher for threat-related pictures than for moral violation pictures conflicts with this interpretation in terms of distinct mechanisms underlying distraction and reappraisal. We suggest another explanation for this result. Indeed the interaction between regulation strategy and emotional content could also be explained by the “distancing” mechanism that underpins the reappraisal strategy. Anger expressions or fear stimulus that signal potential threat are expected to activate avoidant mechanisms (e.g., [48]) whereas distress-related emotions such as sadness or pity conveyed by moral violation pictures could elicit a desire for affiliation or caregiving (e.g., [49, 50]). Thus, reappraisal may be easier to implement for pictures that facilitate avoidance-related behavior than for pictures that facilitate approach-related behavior. Katzir and Eyal [51] showed that self-distancing strategy (corresponding to the reappraisal instruction in our experiment) was not effective in regulating feelings of guilt and shame. The authors explained that self-distancing strategy fail to attenuate self-conscious emotions (such as shame and guilt) because these emotions involve self-evaluation as well as the evaluation of the self from the perspective of others. Given that moral violation pictures in our experiment elicited such self-conscious emotions, it could explain why reappraisal was less effective on this kind of pictures.

In our experiment, reappraisal seems to be more suitable (compared to distraction) for reducing fear or anxiety (induced by threat-related pictures) than for reducing sadness or self-conscious emotions (moral violation pictures). This result, corroborates previous studies where the greatest reduction in threat-related emotions was observed in reappraisal in comparison with the distraction condition (e.g.,[35]). These findings have important clinical implications for the use of distraction and reappraisal in mental health prevention. Indeed, our findings suggest that reappraisal should be preferentially used (compared to distraction) in intervention programs that target anxiety disorders. But regarding on the short-term reduction of sadness, distraction appears to be as effective as reappraisal and can be used as an additional strategy in intervention that target depression symptoms. Nevertheless, current findings should be taken with caution. Indeed, each subject was asked to perform two regulation strategies in a single experimental session. Such design could not avoid the interferences that result from switching among each strategy. Thus, a block design in which only one emotion regulation strategy is included in one block would be more suitable. Current findings need to be replicated with such a design.

Finally, regarding on the effect of social content on regulation strategy effectiveness, results showed no effect of this factor. This result is in contradiction with the findings of Silvers et al. [31] showing that adolescents were less successful in regulating responses of social stimuli than nonsocial stimuli. In our experiment, social pictures were rated as negatively higher than nonsocial pictures, but they were not harder to regulate. However, in the study by Silvers et al. [31], only young adolescents (10 to 13 years old) were less successful at reappraising responses to social stimuli. Adolescents in Experiment 1 were older (14 years old), which could explain the fact that social content does not modulate regulation effectiveness. Indeed, maybe that social factors play a role in regulation more particularly in the transition period to childhood to early adolescence when youths become more sociable, form complex and hierarchical peer relationships and become sensitive to acceptance and rejection by their peers [52]. Consequently, it would be interesting to study several age groups during adolescence in order to specify the effect of social content on regulation strategies.

In conclusion, findings from Experiment 1 strengthen and extend those from previous studies by (1) showing that reappraisal is a highly effective form of emotion regulation (and even more effective than distraction) starting from the age of 14 years, and (2) highlighting distinctions between distraction and reappraisal: reappraisal effectiveness is modulated by emotional content but distraction is not. Reappraisal may thus be optimal in threat-related situations and in the treatment of anxiety symptoms but equivalent to distraction in other emotional situations. However, given the distinctions between distraction and reappraisal [13] and the development of cognitive control abilities and of prefrontal regions that support regulation (e.g., [23, 29], we could hypothesize that these two regulation strategies present different effectiveness at different ages. Moreover, some studies have suggested that instructed use may not be paralleled by increasing spontaneous use with age in everyday life. Experiment 2 examines how regulation strategies effectiveness and their everyday use are modulated by age during adolescence.

## Experiment 2: Are there differences in regulation strategies use and effectiveness in 12-, 13- and 15-year-old adolescents?

The development of cognitive processes and of brain structures in regions subserving emotion regulation suggest that as adolescents mature, they develop more effective emotion regulation. But studies assessing how adolescents regulate their emotions reveal a mixed picture.

Results in which a lab-based paradigm was used suggest development in reappraisal and distraction effectiveness. Indeed, McRae et al. [30], examined reappraisal effectiveness in children (10–13 y/o), adolescents (14–17 y/o) and young adults (18–22 y/o). The authors observed a linear increase in cognitive reappraisal effectiveness with age, which went along with linear increases in the activation of the prefrontal cortex previously associated with reappraisal in adults (e.g., [20]). Silvers et al. [31] confirmed these behavioral results in adolescence from 10 to 23 years old and showed that social factors (i.e., social vs. nonsocial stimuli) interacted with age to predict regulation effectiveness: Young adolescents were less successful in regulating responses of social stimuli than nonsocial stimuli. Conversely, Pitskel et al. 2011 [53] did not find any increases in reappraisal effectiveness to regulate disgust content in 7 to 17 years old. Regarding on distraction, ignoring task-irrelevant emotional distractor stimuli seems to undergo developmental changes during adolescence. For example, negative no-go stimuli lead to an increased false alarm rate for adolescents in comparison to adults [54]. Adolescents also have larger interference effects for negative distractors and overall lower accuracy than adults [55]. Finally, Vetter et al.[28], demonstrate a significant neural developmental step between 14 and 16 years in cognitive control of emotions by an increased activation of top-down control regions when faced with negative emotional distractors. Given these evidences, the ability to daily use and implement effectively distraction and reappraisal strategies should evolve with age in adolescents.

However, studies that have assessed self-reported use of emotion regulation led to mixed results. Gullone, Hughes, King, and Tonge [32] found an overall decrease in the self-reported use of reappraisal strategy in everyday life between the ages of 9 and 15 years. In two recent studies, Lantrip et al. [27] and Sai et al. [56], no age-related differences were observed in the use of reappraisal strategy. On the contrary, Williams and McGillicuddy-De Lisi [57] reported that cognitive change (e.g., reappraisal) showed developmental increases in late adolescence; and Donaldson et al. [33] reported an increase in early and late adolescence, but a decrease in middle adolescence. Regarding distraction strategy, some studies in children and adolescents (e.g., [58]) have found decreases in distraction with increasing age, whereas others suggested increases in the use of cognitive distraction between childhood and adolescence regardless of whether the age range was from 6 to 9, 5/6 to 11/12, 8 to 14, or 10 to 13 years [59].

To better understand these mixed results and the development of emotion regulation abilities during adolescence, Experiment 2 examines age-related differences in regulation effectiveness and in the everyday use of these regulation strategies. Firstly, Experiment 2 examines distraction and reappraisal effectiveness in 12-year-old, 13-year-old and 15-year-old adolescents, using the same paradigm as in Experiment 1. Given the different costs of these two strategies in terms of cognitive resources [13], we hypothesized that younger adolescents with fewer cognitive resources would be less able to implement reappraisal than older adolescents and that the superiority of reappraisal effectiveness (compared with distraction) would be observed only in older adolescents. Secondly, Experiment 2 assesses the everyday use of regulation strategies in 12-, 13- and 15-year-old adolescents by using the Cognitive Emotion Regulation Questionnaire (CERQ; [60]). We aimed to determine whether there are age-related changes in the everyday use of strategies of cognitive change (i.e., reappraisal) and distraction.

## Method

The present experiment was conducted in accordance with the Declaration of Helsinki and the educational organization of France. Informed consent was obtained from all participants included in the study. The ethics committee of the Laboratory of Psychology and Neurocognition from Grenoble-Alpes University and of the Faculty of Psychology and Education Sciences from the University of Geneva approved this study.

### Participants

A total of 172 adolescents from French middle and high school were recruited for this experiment: 59 12-year-olds enrolled in 7^th^ grade, 53 13-year-olds enrolled in 8^th^ grade, and 60 15-year-olds enrolled in 10th grade. Half of the participants were randomly assigned to the distraction strategy group and the other half to the reappraisal strategy group. We choose this between-participants design because even if within-participants designs reduce sampling error and so potentially increase effect sizes, such designs could report larger effect sizes because of demand characteristics (participants may guess that the researcher is comparing instruction X with instruction Y and emphasize the difference either consciously or unconsciously). Moreover this between-participants design can prevent participants from using a strategy other than the one intended during the experimental session (e.g. to reappraise the stimulus when distraction was required). Sample size was determined according to a power analysis (using Gpower) based on a 3 x 2 x 2 x 2 mixed interaction with an α error probability at .05, a power at .90, an effect size at .40 suggesting a total sample size of 156 participants.

About 7.5% of the sample (*N* = 13) were excluded from the analyses were excluded from analyses because of technical problems (N = 3), refusal to terminate the experiment (N = 5) and after statistical analysis of outliers (N = 5). Our final sample included 159 adolescents (79 girls, 80 boys): 56 (30 girls and 26 boys) 12-year-olds (*M* = 12 years and 4 months, *SD* = 5 months), 49 (19 girls and 30 boys) 13-year-olds (*M* = 13 years and 3 months, *SD* = 4 months) and 54 (30 girls and 24 boys) 15-year-olds (*M* = 15 years and 3 months, *SD* = 5 months). The distraction strategy group was composed of 82 adolescents (30 12-year-olds, 26 13 year-olds and 24 15-year-olds) and the reappraisal strategy group of 77 adolescents (26 12-year-olds, 23 13 year-olds and 30 15-year-olds).

The written consent of the parents was obtained for each participant.

### Procedure and material

First, participants were asked to do the regulation task (the same paradigm as Experiment 1). The training phase was the same as in Experiment 1 except that participants were trained to only one regulation strategy (distraction or reappraisal). This was done to prevent them from using a strategy other than the one intended during the experimental session. Of the 160 experimental trials (80 per instructional cue), participants performed the same task as in Experiment 1 except that the reappraisal strategy group saw the cue words “look” or “distance,” whereas the distraction strategy group saw the cue words “look” or “search for.” Two sets of 80 negative images were counterbalanced across participants with look, distraction, and reappraisal instructions. A total of 160 pictures (600 × 400 pixels) were taken from the IAPS [39], the GAPED [42], and a set of similar pictures that had been previously used in research with children [43]. One-half of this set (*N* = 80) depicted “moral violation” pictures and the other half (*N* = 80) depicted “threat-related” pictures. Moral violation pictures are relevant in the elicitation of sadness and pity, whereas threat-related pictures are relevant in the elicitation of fear and anxiety (Experiment 1). Among the set of pictures, one-half (*N* = 80) were social and the other half (*N* = 80) were nonsocial. In sum, the set was composed of 40 social and 40 non-social moral-violation pictures, and 40 social and 40 non-social threat-related pictures. To ensure the valence and arousal of pictures were equal to each other across the three instruction conditions (look, reappraisal, distraction), we created 2 sub-sets of pictures that were counterbalanced across regulation condition and participants. Indeed, half of the participants of distraction group were seeing the first subset (pictures 1 to 20) of each picture category (threat related social, threat related non-social, moral violation social, moral violation non-social) during the look condition. They were seeing the second subset (pictures 21 to 40) of each picture category during the distraction condition. The other half of the distraction group, were seeing the second subset (pictures 21 to 40) of each picture category during the look condition. And they were seeing the first subset (pictures 1 to 20) of each picture category during the distraction condition. The same counterbalancing was used for the reappraisal group. Furthermore, we checked if the valence ratings in passive watching condition were equivalent across the two subsets of pictures (pictures 1 to 20 *vs*. pictures 21 to 40). Results revealed no significant effect of pictures subsets, *F*(1,38) < 1 *p* = .65.

Finally, participants were assessed on their emotion regulation strategies use in everyday life using the The Cognitive Emotion Regulation Questionnaire (CERQ, French version; [61]). The CERQ is a 36-item self-reporting questionnaire designed to evaluate nine cognitive strategies used to regulate emotions in response to negative or unpleasant events [62]. According to Garnefsky et al. [60], acceptance “refers to thoughts of accepting what you have experienced and resigning yourself to what has happened.” Positive refocusing “refers to thinking about joyful and pleasant issues instead of thinking about the actual event.” This strategy can be considered as a subtype of distraction (e.g., [12]). Refocus on planning “refers to thinking about what steps to take and how to handle the negative event.” Positive reappraisal “refers to thoughts of attaching a positive meaning to the event in terms of personal growth.” Putting into perspective “refers to thoughts of playing down the seriousness of the event or emphasizing its relativity when compared to other events.” The two latter strategies can be considered as two subtypes of reappraisal (e.g., [12]). Self-blame “refers to thoughts of blaming yourself for what you have experienced.” Rumination “refers to thinking about the feeling and thoughts associated with the negative event.” Catastrophizing “refers to thoughts of explicitly emphasizing the terror of an experience.” Finally, blaming others “refers to thoughts of putting the blame of what you have experienced on others.” Participants are asked to indicate on a 5-point Likert scale from 1 (*almost never*) to 5 (*almost always*) what they generally think when they experience negative or unpleasant events. Individual subscale scores were obtained by summing the scores belonging to the particular cognitive emotion regulation strategy (ranging from 4 to 20). The higher the subscale score, the more the specific cognitive strategy is used. Acceptance, positive refocusing, refocus on planning, positive reappraisal, and putting into perspective are considered as adaptive strategies, whereas self-blame, rumination, catastrophizing, and blaming others are considered as maladaptive (e.g., [63]). The CERQ is the only regulation questionnaire translated into french and standardized in a sample of french-speaking undergraduates [61]. Adolescents were tested individually in a quiet room within their school.

## Results

### Age-related differences in emotion regulation effectiveness

#### Affect ratings

Mean negative affect ratings were calculated for the look, reappraisal, and distraction conditions. Table 2 presents the mean affect rating according to instruction condition (distraction, reappraisal, look), social content (social, nonsocial), and emotional content (moral violation, threat-related) for the three age groups.

**Table 2:** Mean affect rating (and SD) for 12-, 13- and 15-year-old adolescents according to instruction condition (distraction, reappraisal, look), emotional content (moral violation, threat-related) and social content (social, nonsocial).

		Moral violation	Threat-related	Social	Nonsocial	Total
**12-year-olds**	Distraction	2.99 (0.96)	2.79 (0.99)	3.08 (0.98)	2.70 (0.94)	2.89 (0.91)
	Reappraisal	3.05 (0.95)	2.61 (0.85)	3.07 (0.93)	2.59 (0.93)	2.83 (0.85)
	Look	3.22 (0.98)	3.00 (0.99)	3.35 (0.94)	2.87 (1.03)	3.11 (0.91)
**13-year-olds**	Distraction	2.76 (0.83)	2.99 (0.81)	2.94 (0.79)	2.81 (0.86)	2.88 (0.79)
	Reappraisal	2.82 (0.82)	2.64 (0.87)	2.87 (0.87)	2.59 (0.77)	2.73 (0.78)
	Look	3.05 (0.73)	3.30 (0.85)	3.28 (0.73)	3.07 (0.85)	3.17 (0.73)
**15-year-olds**	Distraction	2.47 (0.75)	2.58 (0.79)	2.72 (0.86)	2.34 (0.72)	2.53 (0.73)
	Reappraisal	2.46 (0.68)	2.15 (0.61)	2.39 (0.73)	2.22 (0.57)	2.30 (0.60)
	Look	2.86 (0.76)	3.00 (0.77)	3.04 (0.80)	2.83 (0.77)	2.93 (0.72)

In order to determine whether the mean affect rating was equivalent according to the different types of pictures, we performed an ANOVA on the mean affect rating in the non-regulation condition (i.e., look), considering emotional content (moral violation, threat-related) and social content (social, nonsocial) as the within-subject factors and age (12-year-olds, 13-year-olds, 15-year-olds) as the between-subjects factor. The negative affect rating was higher for social pictures (*M* = 3.22, *SD* = 0.84) than for nonsocial pictures (*M* = 2.91, *SD* = 0.89), *F*(1, 156) = 31.91, *p* < .001, *partial η*^*2*^ = 0.17. No significant effect of the emotional content factor was found, *F*(1, 156) = 1.03, *p* = .31. Adolescents did not differ on their mean affect rating according to their age, *F*(2, 156) = 1.31, *p* = .27. The social content x age interaction was marginal, *F*(2, 156) = 2.98, *p* = .05, *partial η*^*2*^ = 0.04. A Tukey HSD test revealed that 12-year-olds rated their affect as being more negative after social pictures (*M* = 3.35, *SD* = 0.94) than after nonsocial pictures (*M* = 2.87, *SD* = 1.03, *p* < .001), but this difference was not significant for 13-year-olds (*p* = .23) and 15-year-olds (*p* = .20). In the same way, emotional content significantly interacted with age, *F*(2, 156) = 7.61, *p* < .01, *partial η*^*2*^ = 0.09: In 12-year-olds, affect tended to be rated more negatively after moral violation pictures (*M* = 3.22, *SD* = 0.98) than after threat-related pictures (*M* = 3.00, *SD* = 0.99, *p* = .10), but 13-year-olds tended to rate their affect as more negative after threat-related pictures (*M* = 3.30, *SD* = 0.85) than after moral violation pictures (*M* = 3.05, *SD* = 0.73, *p* = .08) and this difference was not significant for 15-year-olds (*p* = .64). Other interactions were not significant (*F* < 1).

#### Regulation effectiveness

To check for the main effect of regulation (look > reappraisal and look > distraction), we conducted a preliminary ANOVA on these mean negative affect ratings, considering the instruction condition (regulation, non-regulation) as the within-subject factor and regulation strategy (distraction, reappraisal) as the between-subjects factor. Results showed that the mean negative affect was significantly lower when participants were instructed to use regulation strategies (*M* = 2.69, *SD* = 0.80) compared with the non-regulation condition (i.e., look; *M* = 3.07, *SD* = 0.79), *F*(1, 157) = 140.34, *p* < .001, *partial η*^*2*^ = 0.47. The interaction between the instruction condition and regulation strategy was significant, *F*(1, 157) = 25.46, *p* < .001, *partial η*^*2*^ = 0.14. Tukey’s HSD test revealed that the mean negative affect was significantly lower when participants in the reappraisal strategy group used regulation (*M* = 2.60, *SD* = 0.77) compared with the non-regulation condition (*M* = 3.14, *SD* = 0.75). The same effect of regulation instruction was observed in the distraction strategy group (*M*^*D*^ = 2.78, *SD*^*D*^ = 0.82; *M*^*L*^ = 2.99, *SD*^*L*^ = 0.83). The mean negative affect did not differ between the reappraisal and distraction strategy group in the regulation condition (*p* = .74) or in the non-regulation condition (*p* = .82).

The regulation effectiveness score was calculated as in experiment 1. We conducted an ANOVA on regulation effectiveness, considering the regulation strategy group (reappraisal, distraction) and age group (12-year-olds, 13-year-olds, 15-year-olds) as the between-subjects factors, emotional content (moral violation, threat-related) and social content (social, nonsocial) as the within-subject factors. Results showed a significant effect of the regulation strategy factor, *F*(1,153) = 22.42, *p* < .001, *partial η*^*2*^ = 0.13, revealing that regulation effectiveness was higher in the reappraisal condition (*M* = 16.14%, *SD* = 15.57) than in the distraction condition (*M* = 6.82%, *SD* = 8.34). Results also revealed a significant effect of age, *F*(2, 153) = 7.16, *p* < .001, *partial η*^*2*^ = 0.09. Tukey’s HSD test revealed that regulation effectiveness was higher in 15-year-olds (*M* = 16.50%, *SD* = 15.61) than in 13-year-olds (*M* = 10.90%, *SD* = 12.47) (*p* < .05) and 12-year-olds (*M* = 7.06%, *SD* = 9.52) (*p* < .001) but that no difference were observed between 12- and 13-year-olds (*p* = .23). Results showed a significant regulation strategy × age interaction, *F*(2, 153) = 4.24, *p* < .01, *partial η*^*2*^ = 0.05. Indeed, Tukey’s HSD test revealed that regulation effectiveness in 15-year-olds was higher for reappraisal (*M* = 23.27%, *SD* = 15.59*)* than for distraction (*M* = 8.05%, *SD* = 10.98, *p* < .001), that this difference was marginal in 13-year-olds (*M*^*R*^ = 15.83%, *SD*^*R*^ = 15.43; *M*^*D*^ = 6.54%, *SD*^*D*^ = 6.85, *p* = .06) and that this difference was not significant in 12-year-olds (*M*^*R*^ = 8.19%, *SD*^*R*^ = 11.73; *M*^*D*^ = 6.07%, *SD*^*D*^ = 7.17, *p* = .98). Distraction effectiveness was not modulated by age (*p* < .99) while there is a linear increase with age in reappraisal effectiveness (*p* < .01).

Finally to check if distraction was successfully used, we analyzed performance at the distraction task. With a mean percentage of correct response of 83%, results confirmed that participants successfully used distraction.

**Fig 2:**
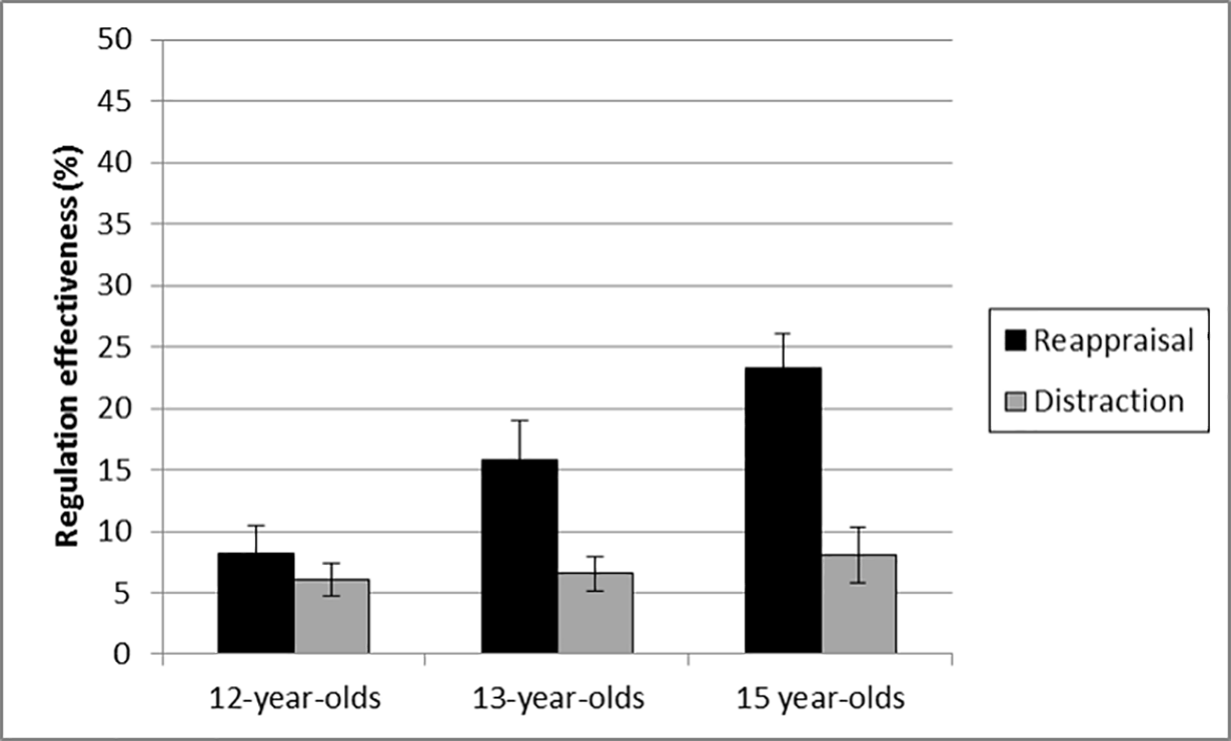
Regulation effectiveness as a function of regulation strategy and age. The error bars correspond to one standard error.

Fig 2 presents regulation effectiveness as a function of regulation strategy and age.

Regarding emotional content, results showed that regulation effectiveness was higher for threat-related pictures (*M* = 13.85%, *SD* = 17.06) than for moral violation pictures (*M* = 9.05%, *SD* = 11.76), *F*(1, 153) = 29.78, *p* < .001, *partial η*^*2*^ = 0.16. The effect of social content on regulation effectiveness was not significant, *F*(1, 153) < 1, *p* = .81. The social content x age interaction, *F*(2, 153) = 2.70, *p* = .07, and the emotional content × age interaction, *F*(2, 153) = 1.36, *p* = .26 were not significant.

Finally, results showed that regulation strategy significantly interacted with emotional and social content, *F*(1, 153) = 40.37, *p* < .001, *partial η*^*2*^ = 0.21, and *F*(1, 153) = 4.69, *p* < .05, *partial η*^2^ = 0.03, respectively. Tukey’s HSD test revealed that regulation effectiveness was higher for reappraisal (*M* = 21.39%, *SD* = 19.09) than for distraction (*M* = 6.41%, *SD* = 10.44) for threat-related pictures (*p* < .01), but not for moral violation pictures (*M*^*R*^ = 10.90%, *SD*^*R*^ = 13.67; *M*^*D*^ = 7.21%, *SD*^*D*^ = 9.24, *p* = .91). Again, reappraisal was less effective with moral violation pictures than with threat-related pictures, *p* < .01, but no significant difference was observed for distraction, *p* = .91.

In the same way, regulation effectiveness was higher for reappraisal (*M* = 17.09%, *SD* = 16.90) than for distraction (*M* = 5.58%, *SD* = 10.56) for nonsocial pictures (*p* < .01), but this difference was not significant for social pictures (*M*^*R*^ = 15.21%, *SD*^*R*^ = 16.32; *M*^*D*^ = 8.05%, *SD*^*D*^ = 9.58, *p* = .15).

### Age-related differences in the everyday use of emotion regulation strategies

Mean scores and standard deviations of the reported use of regulation strategies in the 12–13- and 15-year-old adolescents are shown in Table 3.

**Table 3:** Differences in reporting of regulation strategies between 12-year-old, 13-year-old and 15-year-old adolescents: means, standard deviations, and F-tests.

	12-year-olds		13-year-olds		15-year-olds		Univariate *F*-tests	
	*M*	*SD*	*M*	*SD*	*M*	*SD*	*F*(2, 156)	*p*
Acceptance	12.91	3.72	13.33	3.88	13.11	3.52	<1	.85
Positive refocusing	12.25	4.43	11.90	4.65	9.98	4.22	4.10	< .05
Refocus on planning	12.66	4.01	13.06	3.61	13.22	3.36	<1	.75
Positive reappraisal	13.70	3.63	11.82	4.12	12.83	3.66	<1	.81
Putting into perspective	13.7	3.62	13.53	3.55	12.66	3.84	<1	.49
Self-blame	9.8	2.85	10.61	3.19	10.94	3.12	2.04	.13
Rumination	10.88	3.48	12.55	3.43	12.57	3.86	3.99	< .05
Catastrophizing	9.61	3.41	9.61	3.95	8.74	3.42	1.04	.35
Blaming others	7.89	3.01	8.94	3.46	7.96	2.31	2.00	.13

Overall, regulation strategies differ in their mean reported use, *F* (8, 1264) = 46.69, *p* < .01, *partial η*^2^ = 0.23. Tukey HSD test revealed that the mean frequency of use did not differ between putting into perspective (*M* = 13.35, *SD* = 3.61), acceptance (*M* = 13.11, *SD* = 3.68), refocus on planning (*M* = 12.97, *SD* = 3.66), but that these strategies were more frequently used than rumination (*M* = 11.97, *SD* = 3.67), positive reappraisal (*M* = 11.94, *SD* = 3.59), positive refocusing, (*M* = 11.37, *SD* = 4.52), self-blame (*M* = 10.44, *SD* = 3.07), catastrophizing (*M* = 9.31, *SD* = 3.59), and blaming others (*M* = 8.24, *SD* = 2.97).

We performed MANOVAs on the subscale mean scores, considering age (12-year-olds, 13-year-old, 15-year-olds) as the between-subject factor. Significant overall differences exist between the three groups of adolescents, *F*(18, 290) = 1.65, *p <* .05, *partial η*^*2*^ = 0.09. To study which of the nine cognitive coping strategies were responsible for the significant differences between adolescents, we performed univariate *F*-tests. Results revealed significant age differences for positive refocusing, *F*(2,156) = 4.10, *p* < .05, *partial η*^*2*^ = 0.05, and rumination, *F*(2,156) = 3.99, *p* <. 05, *partial η*^*2*^ = 0.05. Tukey HSD test was performed to determine significant differences. The 12- and 13-year-olds reported more positive refocusing than did the 15-year-olds (respectively *p* < .05 and *p* = .07) but they did not differ from each other on this strategy use (*p* = .91). The 12- year-olds reported less rumination strategies than did the 13- (*p* < .05) and 15 year-olds (*p* < .05) but the two last one did not differ on their rumination use (*p* = .99).

Regarding on the mean frequency use of maladaptive (self-blame, rumination, catastrophizing, blaming others) and adaptive strategies (acceptance, positive refocusing, refocus on planning, positive reappraisal, putting into perspective), results revealed that adolescents more frequently use adaptive strategies (*M* = 12.55, *SD* = 2.74) than maladaptive ones (*M* = 9.99, *SD* = 2.19), *F*(1,155) = 95.01, *p* < .01 *partial η*^*2*^ = 0.38. The mean frequency use of maladaptive and adaptive strategies did not differ according to age, *F* (2,155) = 1.88, *p* = .16.

Regarding on the mean frequency use of cognitive change (i.e., mean frequency of use of positive reappraisal and putting into perspective) and distraction strategies (i.e., mean frequency of use of positive refocusing), results revealed that strategies of cognitive change (*M* = 12.65, *SD* = 3.18) was more frequently used than strategy of distraction (*M* = 11.37, *SD* = 4.52), *F*(1,155) = 13.46, *p* < .01 *partial η*^*2*^ = 0.08. The mean frequency use of cognitive change and distraction strategies did not differ according to Age, *F* (2,155) = 2.42, *p* = .09.

Finally, to determine if the daily use of emotion regulation strategies was linked to the strategies effectiveness, we performed correlational analyses between regulation effectiveness score and the reported use of cognitive change and attention deployment strategies. Results revealed that reappraisal effectiveness did not correlate with the reported use of putting into perspective strategy, *r* = .005, *p* = .96, positive reappraisal, *r* = .10, *p* = .34, or positive refocusing, *r* = .13, *p* = .26. In the same manner, distraction effectiveness did not correlate with positive refocusing, *r* = .32, *p* = .78, putting into perspective strategy, *r* = .05, *p* = .67, or positive reappraisal, *r* = .12, *p* = .31.

## Discussion

First, the findings from experiment 2 replicated those of experiment 1 by showing that unlike distraction, reappraisal was more effective at regulating threat-related pictures than moral violation pictures. This result is discussed in the discussion section of experiment 1. Results showed no interaction between regulation strategy, emotional content, and age. This result seems to run counter to the notion that emotion regulation develops in an emotion-specific manner. For instance, [38] showed a decline in adaptive emotion regulation from early adolescence to middle adolescence for sadness and anger but not for fear. However, studies showing age differences in the use or effectiveness of emotion regulation strategies according to specific emotions [37, 38] have assessed emotion regulation in a more naturalistic context by using self-reported measures. Regarding social content, results showed that reappraisal was more effective than distraction but only for nonsocial pictures but that strategies effectiveness were not modulated by social content. Social pictures were rated as more negative than nonsocial pictures, especially in 12-year-olds. Results showed that adolescents, especially younger adolescents, were particularly sensitive to social scenes, but not less able to downregulate their negative affect using reappraisal in this context. Social factors may play a role in the regulation effectiveness in young adolescents with high rejection sensitivity [31]. Studies that examine the effectiveness of different regulatory strategies in specific situational contexts could lead to the finding of work tools to teach regulatory skills in social contexts for individuals who tend to perceive social information in a negative way.

Second, our results revealed that both distraction and reappraisal strategies were effective in 12-, 13-, and 15-year-olds to downregulate negative affect. This finding reveals the feasibility of coaching youth to use these strategies. Moreover, our findings were in line with the literature that tested adults and suggested that reappraisal strategy was more effective than distraction in diminishing negative affect rating (e.g., [12]). Such evidence emphasizes the importance to integrate this strategy in programs of mental health prevention.

Moreover, our results were in line with the hypothesis that as adolescents mature, they have more effective regulation of emotions, because higher regulation effectiveness was observed in older adolescents (15- years-olds) than in younger adolescents (12-, 13- year-olds). According to our results, there seems to be a shift in the regulation effectiveness during the age period of early to middle adolescence (ages 13 to 15). Findings corroborate previous observations (e.g., [10]) and lead to consider this age period as a key moment in adolescence to coach youth to use regulation strategies.

In addition, regulation effectiveness was equivalent for the two strategies in 12-year-olds, whereas a large improvement in regulation effectiveness was observed from 13- to 15-year-olds for the reappraisal strategy compared with distraction. This finding thus confirmed that the reappraisal strategy effectiveness to downregulate negative affect develops throughout adolescence [30, 31]. Two factors could explain this pattern. First, it may be that older adolescents simply have more experience with reappraisal than do younger adolescents [16]. However, experiment 2 failed to reveal age differences in the self-reported use of reappraisal and revealed that reappraisal effectiveness was not correlated with the frequency of use of reappraisal in everyday life. Second, the improvement in reappraisal effectiveness could be explained by the cerebral maturation in prefrontal regions associated with the improvement of cognitive control abilities over the course of adolescence [23, 29]. In this case, cognitive control training (e.g., working memory) could help regulation development [64, 65] starting at early adolescence and prevent dysfunctional regulation. However, concurrent assessment of cognitive control abilities would have been required to understand the real causes of the better reappraisal effectiveness in older adolescents in the present study. Conversely, the distraction effectiveness was not modulated by age. Cognitive control abilities to ignore task-irrelevant emotional distractor develop between 14 to 16 years of age [28]. Thus, it is possible that our task was too costly in terms of cognitive control to reveal developmental changes in adolescents of 12 to 15 years of age. Moreover, adolescents seem to be specifically sensitive toward distracting emotional stimuli [54, 55]. Previous studies using implicit emotional task, observed consistently that adolescents in middle or late puberty showed increased emotional reactivity to negative stimuli compared to young adults [66, 67] or pre-early adolescents [68, 69] despite engagement in a non-emotional distracting task. These findings can explain why distraction is less effective than reappraisal in regulating negative emotion during adolescence. However, given that these studies suggest that emotional reactivity is strongly impacted by puberty-related effects, it may be fruitful for future work to include pubertal status assessment to examine whether age and pubertal status exert differential effects on emotional reactivity and regulation success.

Results thus revealed that although distraction effectiveness remained steady from the age of 12 to 15 years, abilities to reappraise improved with age. However, some studies have suggested that instructed use may not be paralleled by increasing spontaneous use with age in everyday life. Regarding on the everyday use of regulation strategies, results are in line with those of previous studies showing that the theoretically more adaptive strategies were used more often than the less adaptive strategies in adolescents (e.g., [63]). Adolescents from 12 to 15 years of age thus reported more often using strategies that yielded negative relationships with depression and anxiety (e.g.,[70, 71]). However, results revealed that older adolescents more often reported rumination than did younger adolescents. Moreover, the use of distraction (i.e., positive refocusing) diminished between 12 and 15 years of age. This result corroborates that of previous studies showing decreases in distraction with increasing age (e.g., [58]). Thus, the current results indicating decreased adaptive coping strategies (distraction) and increased maladaptive coping strategies (rumination) among older adolescents suggest an increased risk of developing depressive symptoms in this population [37, 71, 72]. Indeed, high levels of rumination in early and middle adolescence are associated with greater likelihood of experiencing the onset of a future major depressive episode [73]. Furthermore, the response styles theory suggests that distraction is the adaptive alternative to rumination [74]. Several studies have shown that distraction is negatively correlated with depression symptoms [70] and an effective strategy to reduce depressed affect in adolescents [17]. Thus, the decrease in distraction use and increase in rumination use in the transition period of early to middle adolescence can explain the fact that depression symptoms rise drastically from middle adolescence (e.g.,[9]). An important issue for preventive interventions may therefore be to propose approaches that include emphasis on avoiding maladaptive regulation strategies (e.g., stopping rumination in depression) and learning to use adaptive emotion regulation (e.g. the use of reappraisal or distraction in internalized symptoms).

Regarding the reported use of reappraisal and distraction in adolescents, findings extend those from Experiments 1 in showing that adolescents reported using subtypes of reappraisal (i.e., putting into perspective and positive reappraisal) more often than they reported using distraction. Together, the results from these two experiments show that strategies of cognitive change (i.e., reappraisal) are more frequently used in everyday life and are more effective than strategies of attentional deployment (i.e., distraction) in adolescents from 13 years of age. These findings suggest a link between the effectiveness of a strategy and the ability to select it for use in everyday life. However, results failed to found correlation between the self-reported use and effectiveness of regulation strategies. Furthermore, the increase with age in reappraisal is not accompanied by an increase in the reported use of this strategy in everyday life. The current findings seem incompatible with adult findings [75, 76] showing association between reappraisal effectiveness and frequency (i.e., the habitual use of reappraisal). However, to our knowledge this study is the first to combined self-reported and experimental measures of reappraisal frequency and effectiveness across the adolescent age range. The current findings suggest that these abilities may be relatively independent in the sense they don’t develop simultaneously. However, in this study distinctions between regulation frequency and effectiveness are confounded by time scale (frequency measured in the long-term and effectiveness measured in the short-term) and methods (frequency measured with subjective questionnaire, effectiveness measured with affect ratings). Findings in adults have the same methodological limitations [77].

## Limitations and future directions

Although this study sheds new light on emotion regulation abilities in the adolescent’s population, it is important to acknowledge its limitations as well. The first limitation is the absence of manipulation check of strategy implementation. Although we used a between-subjects design to prevent the participants from using a strategy other than the one intended (e.g. to reappraise the stimulus when distraction was required), we cannot be sure of the specific regulation strategy used by participants in each trial. If results at the distraction task seem to indicate that participants successfully used distraction, we did not have manipulation check to verify reappraisal implementation. Without this manipulation check it is complicated to attribute age-related increase in reappraisal to the reappraisal ability (i.e., one's ability to reinterpret or change his/her typical thought for a given emotional event) or to the reappraisal effectiveness (i.e., the extent to which reappraisal regulates emotion to a desirable state). Evidence suggested that reappraisal effectiveness (when strategy is instructed) is independent of individual differences [78, 79]. In light of these findings, age differences in reappraisal may be explained by old adolescents more successful in implementing reappraisal (i.e., reappraisal ability) than younger ones.

To investigate how participants actually implement the instructed strategies, some studies have administered a series of questions at the end of the experiment asking participants to rate the extent to which they used certain strategies to regulate their affect. Such studies showed that because of the demand characteristics of the manipulation checks, participants tend to rate themselves as implementing the strategy they were asked to use to a greater extent than any other strategies (e.g., [80]). Maybe a better manipulation check would have been to ask participants following each picture to rate to what extent they had been able to follow the instructions on emotion regulation they received in the beginning of the experiment (e.g.,[81]). Furthermore, in the absence of manipulation check, it is not clear which reappraisal strategy (imagining that the picture belongs to a movie or imagining standing farther away from the scene) would be responsible for the decrease of negative emotional experience. Although subjects were given examples of typical reappraisal strategies, variability in strategies used may have influenced the current results. Second, results in these two experiments were obtained with the induction of specific strategies instructions. Any other instructions (e.g., reinterpreting situational or contextual aspects of stimuli) could have led to other results. Participants were asked to imagine themselves standing farther away from the scene (self-distancing) in the reappraisal condition, which raises the question of whether any other instructions (e.g., reinterpreting situational or contextual aspects of stimuli) would have led to other results. For instance, Katzir and Eyal [51] suggested that although self-distancing is not effective in regulating self-conscious emotions, other reappraisal strategies that focus on changing the meaning of self-evaluations (e.g., reframing individual belief) might also be effective. Furthermore, for reappraisal, the regulation strategy was self-generated by the participant following instruction whereas it was implicit and part of the task demand for distraction. The visual search asked in the distraction condition can represent an additional cognitive load which research has shown can decrease affective responses to negative stimuli (e.g., [82]). Moreover, active distraction (e.g., explicitly instructing participants to think about something unrelated to the emotion) has been showed to be more effective than passive distraction (e.g., providing a distracting task) [12]. The difference between the development of reappraisal and distraction effectiveness during adolescence must be confirmed with other subtypes of distraction and reappraisal strategies. As noted by Aldao [83], the heterogeneity in the operationalization of regulation strategies is a problematic issue in emotion regulation studies and a way to resolve this issue could consist of the development of a standardized coding scheme to classify regulation instructions.

Third, although the emotion-eliciting stimuli used in experiment 1 have been standardized [39, 42], information lacks about the specific emotion elicited by these stimuli. Experiment 1 showed that threat-related stimuli were judged as mainly eliciting fear and anxiety, whereas moral violation-stimuli were judged as mainly eliciting sadness and self-conscious emotions such as pity. Of note is that the stimuli used in this experiment were judged by adults. However, affective properties of the pictures may be experienced differently from the perspective of an adolescent. Database stimuli specifically standardized to elicit specific emotions and in specific population are needed to pursue investigations on emotion regulation. In the same vein, a limitation of experiment 2 is that the CERQ does not allow us to know which specific situations participants were imagining that they were regulating. It thus does not allow us to know whether some regulation strategies are preferentially used to manage specific emotions triggered by specific situations. Given that the flexible implementation of adaptive strategies in line with contextual demands is associated with better mental health [84], it seems crucial to examine if adolescents can flexibly switch between using different strategies that might be differentially adaptive for varying contexts. Studies suggested that adolescents show some flexibility in emotion regulation because they vary their regulation strategies in relation to the type of stressor (e.g., daily hassles or major life events; e.g., [57]) and specific emotions (e.g., [38]). However, given that experiment 1 and 2 suggest that regulation strategy effectiveness may be emotion dependent, in line with other studies (e.g., [51]), there is a need to determine which regulation strategies are more effective in different contexts and for which specific emotions. Future work should examine regulation strategies effectiveness in various contexts and determine if individuals can flexibly switch between different strategies according to their effectiveness in specific contexts. Such studies have potential implications for clinical or educational interventions that develop regulation skills and emotional learning. Indeed, such interventions could teach adolescents, the flexible use of regulation strategy according to their effectiveness in different contexts. To investigate this emotion regulation flexibility, measuring the implementations of emotion regulation by using daily diary reports, ecological momentary assessments (e.g., [37]), or the experience sampling method (e.g., [85]) would be informative. Indeed, the use of such a design allows for the measurement of numerous strategies by the same person, permitting comparisons of the relative use and effectiveness of strategies both within and across participants in various situations.

Finally, to fully investigate the extent to which discrete emotions matter in emotion regulation, future research needs to incorporate other emotions, both positive and negative. Indeed, this study focused only on the downregulation of negative emotions despite emerging evidence of the importance of regulating states of positive affect [86]. It is often necessary to regulate positive as well as negative states; for example, one may wish to upregulate positive emotions by savoring a recent happy experience, or, conversely, to downregulate positive emotions if they are socially inappropriate.

## General conclusion

Despite its limitations, this study helps fill an important gap in the literature on emotion regulation across this age period and provides new findings regarding age differences and the specificity of emotion in the use and effectiveness of cognitive regulation strategies.

The results of these two experiments contribute to the existing literature on the role of various cognitive regulation strategies in the regulation of affect in an experimentally controlled design. First, findings showed that the reappraisal strategy was more effective than distraction in diminishing negative affect in 13-, 14- and 15-year-olds adolescents. But, results revealed that effectiveness of reappraisal strategy seems to be emotion dependent. Indeed, reappraisal seems to be more suitable for reducing fear or anxiety (induced by threat-related pictures) than for reducing sadness and self-conscious emotions (moral violation pictures). This finding highlights the need to determine which regulation strategies are more effective in different contexts and for which specific emotions/disorders in order to teach adolescents in prevention programs, more particularly in the period of middle adolescence, to use them flexibly in their everyday lives.

Second, our results showed an increase with age in regulation effectiveness and more particularly in reappraisal effectiveness. This increase in reappraisal effectiveness did not, however, coincide with an increase in the frequency of use of this strategy and other adaptive strategy. From our results, even if emotion regulation (at least reappraisal) is more effective in 15-year-olds, their choice of regulation strategies in everyday life is not more effective than that of 12-year-olds. These findings have important implications for mental health prevention in showing that the selection stage of the emotion regulation process (i.e. moment to choice to use a specific regulation strategy) [87] should be a specific target in intervention programs. Such intervention could coach adolescents to develop their regulation repertoire (i.e, to increase regulatory options), to correctly value (adaptive *vs*. maladaptive) regulation strategies, and to develop their self-efficacy beliefs to employ emotion regulation strategies.

Finally, we observed that with increasing age, the adaptive strategy of distraction was less reported and the maladaptive strategy of rumination was more frequently reported. On the basis of this self-reported assessment, older adolescents appear as a population to be more likely to develop internalized problems than younger adolescents. If emotion regulation is a risk factor for future psychopathology, techniques targeting emotion regulation skills should be incorporated into both treatment and preventive interventions.
